# The Influence of Meteorological Conditions and Seasons on Surface Ozone in Chonburi, Thailand

**DOI:** 10.3390/toxics13030226

**Published:** 2025-03-19

**Authors:** Sawaeng Kawichai, Wissanupong Kliengchuay, Htoo Wai Aung, Sarima Niampradit, Rachaneekorn Mingkhwan, Talisa Niemmanee, Wechapraan Srimanus, Walaiporn Phonphan, San Suwanmanee, Kraichat Tantrakarnapa

**Affiliations:** 1 Research Institute for Health Sciences (RIHES), Chiang Mai University, Chiang Mai 50200, Thailand; 2 Department of Social and Environmental Medicine, Faculty of Tropical Medicine, Mahidol University, Bangkok 10400, Thailand; wissanupong.k@gmail.com (W.K.); htoowai.aun@student.mahidol.ac.th (H.W.A.); sarima.nia@student.mahidol.ac.th (S.N.); ruchneekorn.min@mahidol.ac.th (R.M.); srimanusw@gmail.com (W.S.); 3 Environment, Health & Social Impact Unit, Faculty of Tropical Medicine, Mahidol University, Bangkok 10400, Thailand; 4 Faculty of Science and Technology, Suan Sunandha Rajabhat University, Bangkok 10300, Thailand; talisa.ni@ssru.ac.th (T.N.); walaiporn.ph@ssru.ac.th (W.P.); 5 Department of Epidemiology, Faculty of Public Health, Mahidol University, Bangkok 10400, Thailand; san.sua@mahidol.ac.th

**Keywords:** surface ozone, temporal distribution, seasonal variations, generalized additive model

## Abstract

This study aims to examine the relationship between meteorological factors, specifically temperature, solar radiation, and ozone concentration levels. Levels of surface ozone were monitored (O_3_) in Chonburi, Thailand (located at 3.2017° N, 101.2524° E), from January 2010 to December 2020. Thailand’s coastal tropical environment provided a unique setting for the study. The study revealed a distinctive seasonal trend in ozone levels, with the highest concentrations occurring during the winter and the lowest in the rainy season, on average. The increase of O_3_ in the summer was primarily attributed to intense ground-level solar radiation and higher temperatures of around 30–35 °C, enhancing O_3_ concentrations ranging from 200 to 1400. During the winter, there is an increased elimination of the O_3_ concentration by higher levels of NO_2_. The study also examined the relationship between ozone levels and various meteorological factors to identify which had the most significant impact on ozone formation. The analysis showed that the ozone concentration has a strong negative correlation with relative humidity but is positively correlated with solar radiation, temperature, and wind speed.

## 1. Introduction

Ground-level ozone holds significant importance due to its role in the formation of photochemical smog. Unlike other pollutants, ground-level ozone is not directly emitted into the atmosphere. Rather, it develops through chemical reactions involving nitrogen oxides (NOx) and volatile organic compounds (VOCs). The repercussions of ozone are substantial, impacting both public health and the environment. It possesses the potential to damage respiratory tissues, causing inflammation and irritation, often resulting in symptoms such as coughing, chest tightness, and aggravated asthma. Therefore, understanding the key factors contributing to daily fluctuations in ozone levels is crucial for gaining insights into its behavior. Numerous research studies have tackled this issue using various methodologies, analyzing different variables and metrics [1]. The variability in O_3_ concentrations is more pronounced during spring and summer compared to winter. Moreover, the “weekend effect” phenomenon, where O_3_ levels rise on weekends despite lower precursor levels, has been observed in certain American regions since the 1970s [2].

The studies indicated a correlation between increased radiation and temperature and elevated ozone levels on clear days. Additionally, O_3_ levels are influenced by various weather factors, such as morning radiation exposure, time since the last frontal passage, wind speed, and humidity levels [3]. O_3_ concentrations tend to fluctuate more on clear days than on cloudy days. A previous city-level study projected an increase in compound-event days for heatwaves and ozone pollution from 27.5% to 29% for all days between April and September in Germany from 2081 to 2100 [4]. Ozone reacts to variations in the weather and climate, with fluctuations being more noticeable in spring and summer than in winter [5]. Studies conducted in Europe and North America have significantly improved our understanding of ozone chemistry, meteorology, emissions, and their impact on lower atmospheric conditions. Integrating O_3_ measurements at the ground level with weather data improved our understanding of photochemical reactions under different tropospheric conditions [1]. O_3_ pollution has worsened in Northeast China, with variations in concentrations across the region: lower levels in the north and higher levels near the Bohai Rim in the southeast, close to the major pollution hub of North China [6]. Furthermore, the formation of O_3_ is closely linked to the solar intensity, which, in turn, is influenced by the temperature in the atmosphere. Interestingly, an improvement in atmospheric visibility occurred when there was a reduction in the levels of carbon-based aerosols such as dust that were released through the burning of coal, diesel, and biomass. This increase in visibility allows more sunlight to penetrate the atmosphere and promotes the formation of ozone [7]. Moreover, other favorable meteorological conditions such as warm temperatures, gentle winds, and either land or sea breezes at coastal sites significantly influence ozone concentrations [8].

In Thailand, the ground-level ozone concentration was high, usually in summer (February to June), but occurred at safe levels in July to January between 2017 and 2019 in the upper northern part of Thailand [9]. In Bangkok, the concentrations occurred at the maximum from December to April (dry) and minimum from May to October between 2014 and 2015, and variations occurred, with altitude and location relating to different sources, meteorological factors, and solar radiation. In urban areas, the ozone concentration was higher with height, but decreased ozone was found at higher altitudes in rural areas [10]. A study in 2009 estimated that 61,577 disability-adjusted life years (DALYs) due to chronic obstructive pulmonary disease (COPD) were attributed to ozone in Thailand [11], and it has been observed that ozone pollution was responsible for 0.78% of total non-accidental mortality and mainly associated with cardiovascular diseases [12]. Furthermore, it was observed that a 1 ppb increase in ozone concentration was associated with higher Outpatient Department (OPD) visits for acute upper respiratory infections (RR = 0.08) and circulatory disease mortality (RR = 0.27) in the Eastern Economic Corridor (EEC) of Thailand, including Rayong, Chachoengsao, and Chonburi Provinces [13].

Essentially, statistical models can help us to understand, predict, and manage ground-level O_3_ by connecting it to the weather. They can help predict the time and location high O_3_ concentrations may occur. By separating the influence of weather from other factors, they allow scientists to study long-term changes in ozone pollution. Statistical models can give insight into the complex interplay between chemical reactions and meteorological conditions that drive ozone levels [14]. A study of comparison by predicting the daily max 8-h O_3_ with three statistical models established that the generalized additive model (GAM) offered a marginal improvement over the multiple linear regression (MLR) model and principal component analysis (PCA) and regression (PCR) when modeling O_3_ concentrations. This finding indicates that daily variations in the O_3_ concentrations in continental South Africa are largely influenced by regional O_3_ precursors in combination with meteorological factors [14].

Chonburi Province, recognized as a key economic and tourism center in Thailand, is situated in the eastern region along the Gulf of Thailand’s coastline. Over the past century, it has emerged as one of the most ozone-polluted areas in the country. Positioned in the eastern region, Chonburi has undergone considerable urbanization and industrialization, contributing to the proliferation of ozone pollution issues across various cities nationwide [15]. The aim of our study is to explore selected factors that significantly affect the variability of ozone concentrations in Chonburi Province. In an effort to decrease ozone levels, it is of utmost importance to know how temperature and solar radiation affect pollutant levels, potentially offering insights into ozone management and forecasting.

## 2. Materials and Methods

### 2.1. Study Area

**Figure 1:**
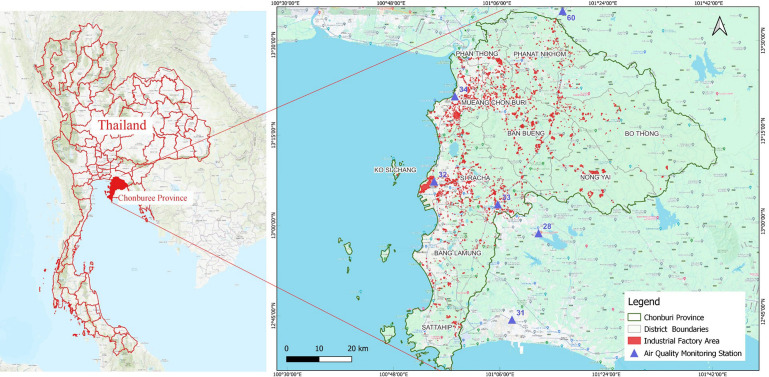
The location of monitoring stations in Chonburi Province and neighboring provinces (QGIS 3.22 Biatowieza) [16,17].

Chonburi Province, as depicted in Figure 1, is located in the eastern part of Thailand and covers an area of 4363 km^2^. It has a population of 1,618,066 people, resulting in a population density of 371 inhabitants/km^2^. The northeast and southwest monsoons predominantly influence the province, mirroring the climates of many other parts of Thailand. The province’s GDP amounts to approximately 16,391 USD, with the industrial sector accounting for more than 50% of this total. The industrial sector covers approximately 2.93% of this province (128 km^2^). It holds a significant role in the economic and tourism sectors of Thailand. Additionally, Chonburi Province is part of the Eastern Economic Corridor (EEC) initiative, which is integrated into Thailand’s 20-year National Strategy. This agenda transforms the eastern region into a hub for advanced manufacturing, innovation, and high-tech industries.

### 2.2. Data Sources

The Pollution Control Department (PCD) in Thailand provides real-time monitoring data on major air pollutants through its network of air quality and meteorological stations. For this study, we used data from Chonburi Province—specifically, utilizing data collected from stations operated by the PCD (ST32, ST33, and ST34). In addition, we also used the data from Chachoengsao and Rayong Provinces to predict the spatial and temporal distributions. Within the urban area, three stations dedicated for monitoring the surface air quality and meteorological conditions (ST-32, T-33, and ST-34) are operated by the Pollution Control Department (PCD), as illustrated in Figure 1 at three stations (ST-28 and ST-31 situated in Rayong Province and ST-60 located in Chachoengsao Province). Typically, various pollutants, including ozone (O_3_), particulate matter (PM), sulfur dioxide (SO_2_), carbon monoxide (CO), nitric oxide (NO), nitrogen dioxide (NO_2_), nitrogen oxides (NOx), total hydrocarbons (THC), methane (CH4), non-methane hydrocarbons (NMHC), and the meteorological parameters, were regularly assessed. However, the data that we applied were ground-level ozone, particulate matter (PM), sulfur dioxide (SO_2_), carbon monoxide (CO), nitrogen dioxide (NO_2_), and the key meteorological parameters of this study.

### 2.3. Spatial and Temporal Distribution Characteristics of the O_3_ Concentration

In this study, the inverse distance weighted (IDW) interpolation method was used to estimate the O_3_ values. The IDW estimated values for unmeasured locations were based on measured points, under the assumption that closer values have greater relevance to the sampling point [18]. Using QGIS [16], O_3_ concentration data was inputted, formatted, and spatially referenced (ST-28, ST-31–ST-34, and ST-60). The ’Interpolation’ tool in the ’Raster’ menu was used to apply the IDW method, with the power parameter set to control the influence of distance based on interpolation. A higher power had more weight at closer locations, while a lower power distributed its influence more evenly. The search radius and number of points examined were optimized to balance local details and broader trends. The IDW algorithm produced a continuous raster surface displaying regional ozone distribution, which was used to analyze spatial patterns, identify high and low concentration areas, and determine potential hotspots.

### 2.4. Statistical Analysis

We employed a generalized additive model (GAM) [1] to investigate the association between ambient ozone concentrations and meteorological factors, using Python (version 3.0) and RStudio Software (Openair Package) (version 2024.12.1) for simulations and analysis from data derived from Chonburi Province (ST-32, ST-33, and ST-34). Descriptive statistics were analyzed by XLstat software (version Basic+ 2023). The same functional form for the GAM model was applied to all seasons to guarantee consistent outcomes. However, we fit the model individually for each season to obtain season-specific characteristics that are logically consistent. The GAM model is set up for a specific season as follows:

(1) $$Y_{t}=\beta_{0}+S_{T}\left({Temp}_{t}\right)+S_{G}\left({GLRD}_{t}\right)+S_{H}\left({RH}_{t}\right)+S_{{NO}_{2}}\left({{NO}_{2}}_{t}\right)+S_{TG}\left({Temp,GLRD}_{t}\right)\;+{\;S}_{TH}\left({Temp,RH}_{t}\right)+S_{GH}\left({GLRD,RH}_{t}\right)+\varepsilon_{t}$$

where:

- $Y_{t}$ is the daily mean ozone concentration for day t (time is indexed by the number of days since the first day of the data), which is an unknown constant (intercept) to be estimated from the data;
- ${Temp}_{t}$ is the mean temperature for day t;
- ${GLRD}_{t}$ is the mean daily global solar radiation (GLRD) for day t;
- ${RH}_{t}$ is the mean relative humidity for day t;
- ${{NO}_{2}}_{t}$ is the mean NO_2_ concentration for day t;
- $\varepsilon_{t}$ is the random error term. We adopted a working assumption of $\varepsilon_{t}$∼N (${0,\sigma }^{2}$) $\varepsilon_{t}$∼N (${0,\sigma }^{2}$), which is a normal, zero mean, homoscedastic distribution of errors;
- $S_{T}$ is an unknown univariate function of temperature, the form of which needs to be determined from the data. We estimated it using a penalized spline, which regulates smoothness by penalizing the integral of the squared second derivative with respect to temperature. This method allows for potentially non-linear, smooth relationships between O_3_ and temperature;
- $S_{G}$ is an unknown univariate function of GLRD to be estimated from the data as a penalized spline;
- $S_{H}$ is an unknown univariate function of humidity to be estimated from the data as a penalized spline;
- $S_{{NO}_{2}}$ is an unknown univariate function of NO_2_ to be estimated from the data as a penalized spline;
- $S_{TG}$ is an unknown bivariate function of temperature and GLRD that needs to be estimated from the data using a tensor product penalized spline. This term enables us to explore the potential interaction between temperature and GLRD in affecting the O_3_ concentration, moving beyond their mere additive effects. Essentially, this effect allows us to investigate how temperature influences the impact of GLRD;
- ${\;S}_{TH}$ is an unknown bivariate function of temperature and RH that needs to be estimated from the data using a tensor product penalized spline. This term represents the interaction between temperature and RH in a parsimonious formulation;
- $S_{GH}$ is an unspecified bivariate function of GLRD and RH, which will be estimated from the data using a tensor product penalized spline. This term represents the interaction between GLRD and RH in a parsimonious manner.

### 2.5. TROPOspheric Monitoring Instrument (TROPOMI)

TROPOMI tropospheric O_3_ data were obtained from the Sentinel-5P satellite, which provides high-resolution offline imagery of atmospheric ozone concentrations. This dataset is crucial for assessing temporal variations in ozone levels, and in this study, the total column O_3_ data were used to analyze atmospheric ozone changes within the study area. The dataset spans from January to December 2019 in Chonburi Province, ensuring comprehensive seasonal coverage, as 2019 represents the period of study following the initial availability of Sentinel-5P data in 2018. To evaluate ozone distribution patterns, the dataset was processed using the Google Earth Engine (GEE) platform, which provides access to pre-processed Sentinel-5P Level-3 data. The dataset that was used, COPERNICUS/S5P/NRTI/L3_O_3_, was filtered based on time (2019) and space parameters that were important to the study area. This made sure that the measurements of ozone were accurate and specific to the area for further study.

## 3. Results

### 3.1. The Spatial–Temporal Distribution of the O_3_ Concentration and Seasonal Variations

#### 3.1.1. The Spatial–Temporal Distribution of the O_3_ Concentration

**Figure 2:**
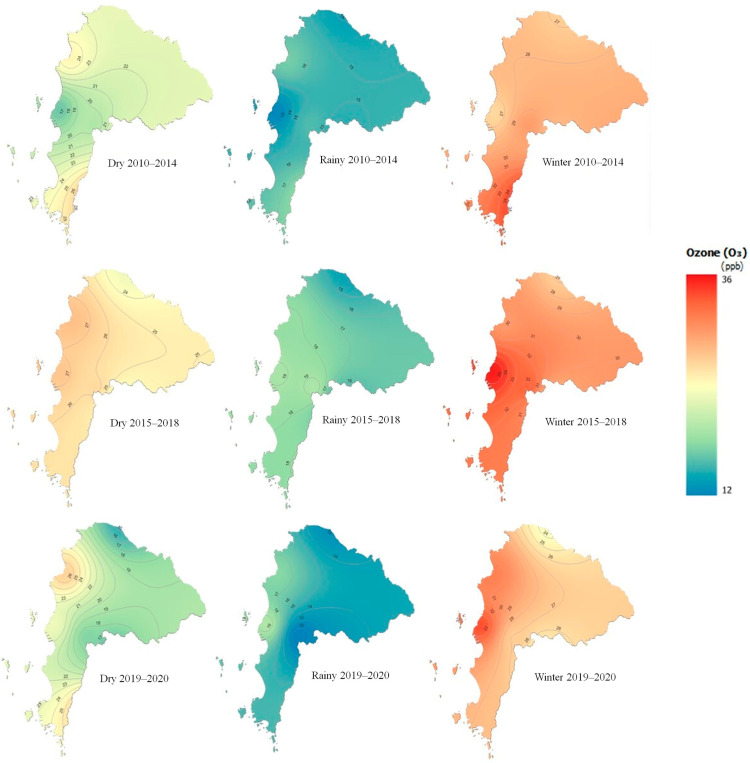
Monthly average concentration spatial distribution of the seasonal average O_3_ in Chonburi Province during 2010–2014, 2015–2019, and 2019–2020 (endemic COVID-19).

**Figure 3:**
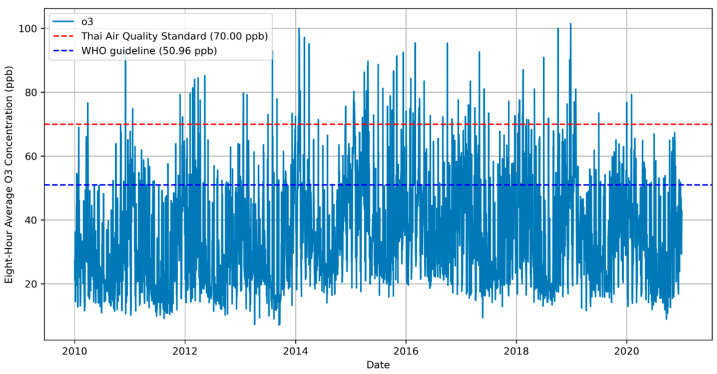
The eight-hour average O_3_ concentrations measured from 9:00 to 17:00 over 10 years from 2010 to 2020.

Figure 2 illustrates the seasonal variations in Chonburi Province in 8-h ozone concentrations, which showed significant shifts throughout the seasons. The illustrations of the geographical distribution of ozone for all studied year ranges (2010–2014, 2015–2018, and 2019–2020) suggest that the winter seasons often have greater O_3_ concentrations compared to the rainy (mid-May to mid-October) and dry (mid-February to mid-May) seasons. Increased photochemical ozone formation, warmer temperatures, and less rain scavenging during the dry months are some possible factors for these shifts. These results indicate that high concentrations occurred during the winter season (mid-October–mid February) from 2010 to 2014. Similarly, the highest concentration was observed during the winters of the 2015–2018 period. The variation in O_3_ concentration has been steady from 2010 to 2019–2020, despite the impact of the COVID-19 pandemic across Thailand in the latter periods. Figure 3 displays the eight-hour average ozone concentrations, measured from 9:00 am to 5:00 pm, over a 10-year period from 2010 to 2020. The concentrations are plotted as a time series, with the vertical axis representing the ozone concentration in parts per billion (ppb) and the horizontal axis representing the years. The data, represented by a solid blue line, show significant variability over time, with some periods experiencing higher average concentrations that result in distinctive spikes above the overall trend. Figures S1 and S2 illustrate time variation plots for other air pollutants and meteorological parameters.

The illustration demonstrates that the ozone concentrations fluctuate frequently, exceeding the WHO guidelines, with several instances surpassing the Thai standard. This pattern indicates that recurring periods when the air quality poses a concern are based on the ozone levels. The presence of variability and spikes may be associated with specific environmental events, industrial activities, or other factors influencing the ozone levels. The data point spread indicates days with relatively stable ozone levels, as well as those with high variability. within the eight-hour window.

#### 3.1.2. Diurnal and Seasonal Variations

**Figure 4:**
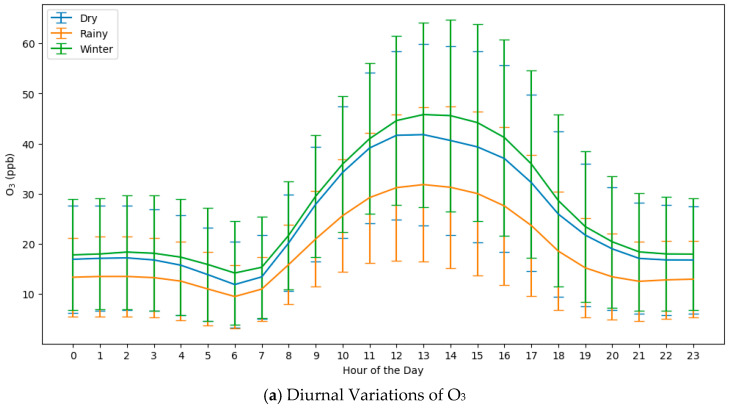

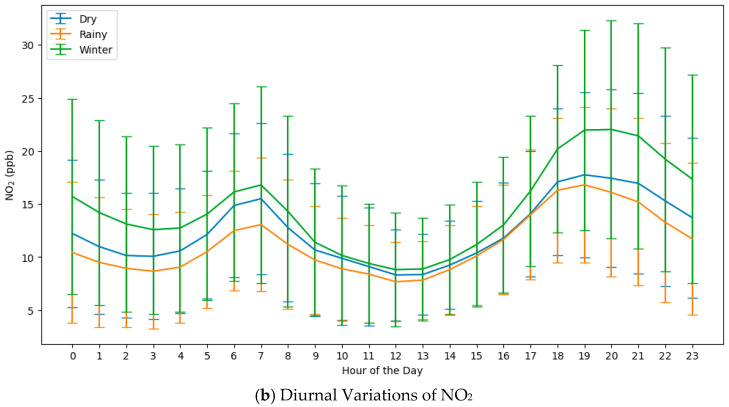
The diurnal (daily) variations of (**a**) the O_3_ and (**b**) NO_2_ levels across different seasons in Chonburi Province from 2010 to 2010.

The model utilized data from the years 2010 to 2020. Table S1 provides descriptive statistical results, including count, mean, standard deviation, minimum value, 25th percentile, 75th percentile, and maximum value (max), for the daily parameters separated into three seasons. The ozone levels are plotted against the hours of the day, as provided in Figure 4, with each season representing a different color, such as blue for dry, orange for rainy, and green for winter. According to the diurnal pattern for all three seasons, the ozone levels are lowest during the early morning hours, begin to rise after sunrise, peak in the afternoon, and then decline toward the evening. It also indicates the standard deviation of the ozone levels at each hour, providing a sense of the variability in the measurements. The length of these bars shows that there is more variability in the ozone levels at certain times of the day, notably in the afternoon hours when they are the highest. The timing of the peak ozone levels appears to be mid-afternoon for all seasons, specifically between 13:00 and 15:00.

This cycle is characteristic of the ozone concentrations, which are formed by photochemical reactions induced by sunlight. Hence, the levels rise after sunrise, peak when solar radiation is at its maximum, and fall after sunset. The winter season shows the highest peak of the average ozone levels, which could be due to more intense solar radiation and possibly lower relative humidity during this season. The dry season represents the second highest levels, while the rainy season shows the lowest, on average. Surprisingly, the second-highest peak occurred in the rainy season. The lower levels in the rainy season may relate to the reduced intensity of solar radiation and potentially higher relative humidity. Focusing on the lowest and peak ozone concentrations, the lowest ozone level occurred around 6 AM, started to rise again at 7 AM, and peaked at around 1 PM in Chonburi Province. The diurnal cycle of NO_2_ was shaped like double waves (Figure 4b). The morning peak was lower in magnitude than the evening peak. The morning peak of NO_2_ was 1–2 h, and the O_3_ peak appeared about 7 h after the NO_2_ morning peak. After the evening peak (7:00 pm), NO_2_ slightly decreased until it reached its lowest value. The NO_2_ decrease correlated with an increase in O_3_.

### 3.2. Association of O_3_ with Another Pollutant and Meteorological Parameter

#### The O_3_ Correlation with Another Factor

**Figure 5:**
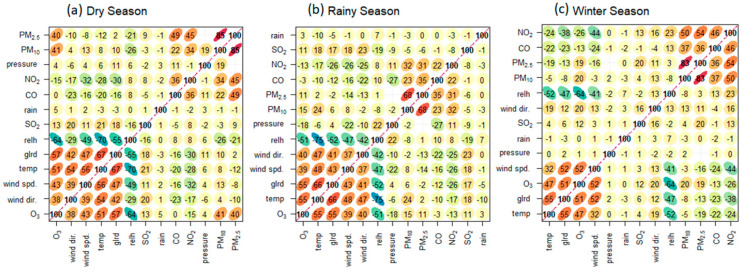
The Spearman’s rank correlation coefficients between the O_3_ levels and a range of other environmental factors in Chonburi Province during the (**a**) dry season, (**b**) rainy season, and (**c**) winter season.

Location-specific factors and the time period studied may also influence the ozone concentration (Figure 5). A strong negative correlation of 0.59–0.69 indicates that O_3_ concentrations decrease as relative humidity increases across all three seasons. A positive correlation of 0.53–0.57 suggests that the higher global radiation is directly associated with the higher O_3_ concentrations. This study found that a moderate positive correlation of 0.48–0.59 suggests that higher temperatures may be associated with increased levels of ozone. Also, there is a moderate positive correlation of 0.52–0.61 influenced by varying wind speeds. The higher wind speeds can contribute to the dispersion of O_3_ and the precursors involved in ozone formation. The direction of the wind has a big effect on the amount of O_3_ in the air during the dry and wet seasons (0.46–0.49), especially near the coast (Figure S3). The wind alters the rates of photochemical formation and the flow of oxygen. Weak positive correlations were observed between O_3_ and PM_2.5_ (0.15–0.35) and O_3_ and PM_10_ (0.19–0.38) in the winter and dry season, respectively. Although these correlations are positive, suggesting some relationship between higher particulate levels and higher ozone levels, the mechanism is not straightforward. However, the seasonal correlation plot highlights both positive and negative correlations between these variables. The colors in the correlation matrices show the direction and strength of correlations as green to blue means negative correlations (blue being the strongest), and red to yellow means positive correlations (red being the strongest). The purple line indicates that the pivotal in understanding the interactions between radiation, relative humidity, and NO_2_ and CO concentrations in the air quality and meteorological dataset.

### 3.3. The Effect of the Interactions of Meteorological Parameters

#### The Interactions Influencing the Ozone Level

**Figure 6:**
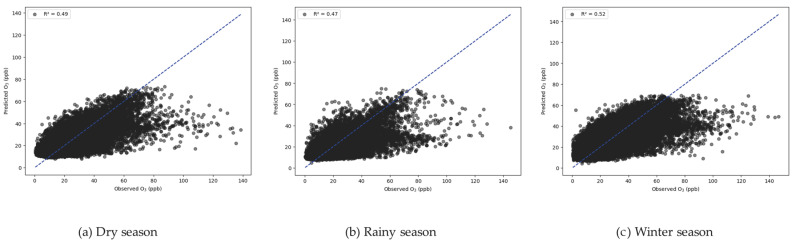
The scatter plot comparing observed and predicted O_3_ concentrations: (**a**) dry season, (**b**) rainy season, and (**c**) winter season.

**Figure 7:**
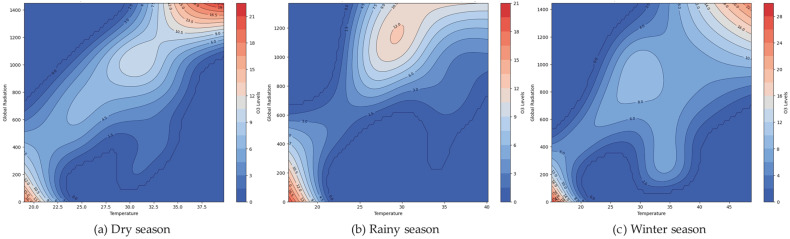
Interaction between GLRD and temperature on the O_3_ concentration in Chonburi Province: (**a**) dry season, (**b**) rainy season, and (**c**) winter season.

**Figure 8:**
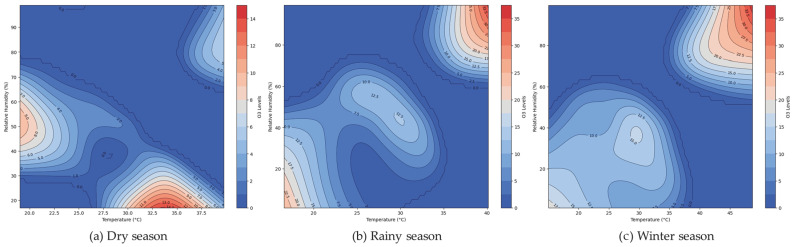
Interaction between relative humidity and temperature on the O_3_ concentration in Chonburi Province.

**Figure 9:**
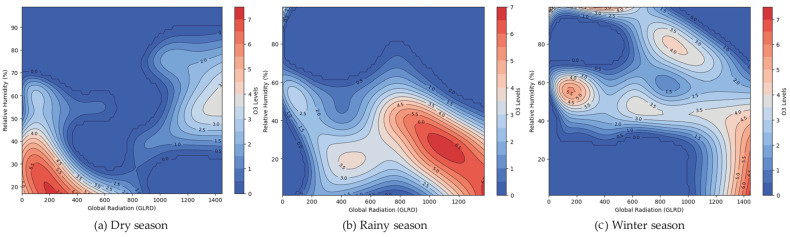
Interaction between relative humidity and GLRD on the O_3_ concentration in Chonburi Province.

According to the generalized additive model (GAM), Figure 6 presents the scatterplot that depicts the relationship between observed and predicted O_3_ concentrations for the three seasons. The O_3_ values that are observed are on the x-axis, while the predicted O_3_ values are on the y-axis. With an R^2^ value between 0.47 and 0.52, the fit is moderate, which means that the GAM equation of three seasons can explain the 47–52% difference in the O_0_ concentrations. The model that includes the effects of air temperature, global radiation (GLRD), relative humidity, and NO_x_ concentration, as well as the interactions between temperature × GLRD, temperature × relative humidity, and GLRD × relative humidity, does a good job of showing how ozone levels change from day to day. Figure 7, Figure 8 and Figure 9 illustrate the smooth terms with 95% confidence intervals derived from the GAM model for Chonburi Province (Table 1). The model complexity is indicated in each season by the estimated degrees of freedom (EDF) and reference degrees of freedom (Ref.df), while the F statistic shows the strength of the association between each factor and the dependent variable. The whole factor and the seasons have extremely low *p*-values (<0.001), indicating statistical significance in these associations. The interaction terms, particularly the NO_2_ effect in the winter and the Temp × RH interaction in the rainy season, have very high F values, indicating large, combined impacts on the dependent variable during these times. Overall, these results highlight that each factor significantly affects the dependent variable, and these effects are consistent across different seasonal conditions. The variance explained for all seasons is estimated to be around 50%.

Figure 7 displays the peaks (areas of high O_3_ levels) in this province, where the GLRD values range from 200 to 1400, with temperatures between 30 and 35 °C. In contrast, when the global radiation and temperatures are decreased, the ozone levels are often reduced over all three seasons (around 15–20 °C).

Figure 8 illustrates the interaction between temperature and relative humidity affecting the O_3_ levels during the dry, rainy, and winter seasons. The gradient in the dry season graphic, which shifts from blue (lower O_3_ levels) to red (higher O_3_ levels), illustrates that the ozone levels rise as the temperature rises and relative humidity falls. However, the interaction pattern in the rainy season increased the humidity, and moderate temperatures resulted in lower ozone levels. The winter season shows a pattern of low temperatures and varying humidity, leading to moderate ozone concentrations.

Figure 9 demonstrates the influence of the relationship between relative humidity and GLRD on the O_3_ concentrations over the dry (Figure 9a), rainy (Figure 9b), and winter (Figure 9c) seasons. The gradient in the dry season graphic, changing from blue (indicating lower O_3_ levels) to red (indicating higher O_3_ levels), indicates that ozone levels decrease as the relative humidity exceeds 60% during the dry and rainy seasons; conversely, in winter, the O_3_ levels are more concentrated within a narrow range of GLRD (~100–300 W/m^2^) and relative humidity (40–50%).

### 3.4. The Spatial Distribution of the O_3_ Concentration

**Figure 10:**
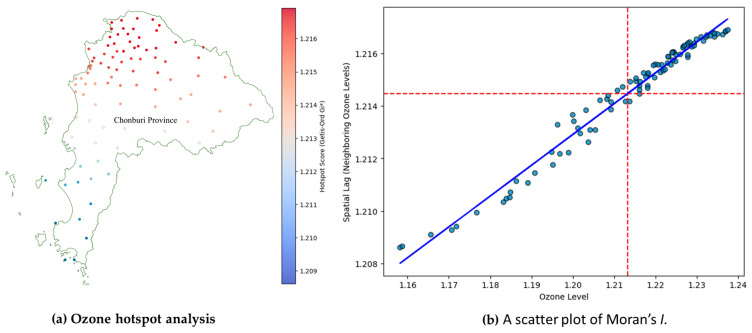
The spatial distribution of the O_3_ concentration (**a**) spatial clustering method using Getis–Ord Gi* and (**b**) Moran scatterplot showing spatial autocorrelation.

Figure 10 presents the spatial autocorrelation of the O_3_ concentration among provinces in Chonburi Province. We performed a spatial autocorrelation analysis on the O_3_ concentration from 2018 to 2019. The map shows the study of Chonburi Province’s high-risk areas (hotspots) for ozone levels using spatial statistical methods like Getis–Ord Gi*, a tool used to find places where values are much higher or lower than the average. The hotspot scores found that the range was 1.209 to 1.216, representing ozone levels in each area.

The Moran’s I value is 0.1175, which means that there is a small positive spatial autocorrelation of the ozone concentration in this province. This means that places with similar levels of ozone tend to be close to places with high levels of ozone (Figure 10a).

## 4. Discussion

Comparing the year ranges, the 2015–2018 period seems to exhibit relatively higher ozone levels across all seasons as compared to 2010–2014 and 2019–2020. This might be related to changes in the meteorological patterns or air quality management strategies implemented during that time frame. Another possible factor is due to the deviations in emission levels associated with the COVID-19 pandemic in 2020. Thailand’s first case was reported in January 2020, and a national lockdown was announced in April 2020 [19]. Moreover, the elevated surface ozone levels in Chonburi are likely driven by industrial activities and heavy traffic. These petrochemical complexes produce volatile organic compounds (VOCs) and nitrogen oxides (NOx), the key precursors for ozone formation. Traffic congestion, particularly during rush hours, increases NOx emissions from vehicles. Sea breezes transport these pollutants inland, creating favorable conditions for ozone production, while proximity to the Gulf of Thailand may facilitate pollutant transport from other coastal areas [15,20].

The results show similarities with previous studies on Southwest Asia [20] and China that observed the average ozone concentration exceeding the WHO guidelines by over 40 days in each year from 2014 to 2017 in Beijing, China. However, the suburban areas exhibited the highest pollutions levels, exceeding those observed in urban and high traffic areas [21]. For the variations and spikes, a study in South Korea presented that the increased domestic emission of VOCs and persistent NO_x_ emission determined the variations and peaks during summertime [22]. Furthermore, a nationwide study using satellite data in China presented that the ozone concentration was influenced mainly by human activities in highly urbanized areas but meteorological factors and agricultural regions in low urbanized areas [23].

Globally, around 12% of cities are facing high ozone pollution, with the highest concentration occurring in summer. Likewise, the results can be compared to the findings from a study in Guangzhou, where the lowest concentration occurred around 7 AM, while the highest levels were observed around 4 PM. At 7:00 AM, sunlight initiates a sequence of photochemical reactions. NO is transformed into NO_2_ through a reaction with O_3_. During the daytime, NO_2_ is converted back to NO by photolysis, hence increasing the regeneration of O_3_ [24].

The meteorological factors, including temperature, humidity, and wind speed, are the main driving factors for seasonal variations [25]. The results are comparable with the studies from China. In the summer of 2019, the ozone concentrations were the highest across China, with particularly elevated levels in Beijing in 2022. In contrast, the highest concentrations occurred in autumn in Guangzhou and in spring in Shanghai, while winter consistently showed the lowest levels across these regions [6,7,26,27]. The studies suggested that the ozone concentration positively correlates with solar radiation and may be a contributing factor to its highest concentration during the dry seasons. In terms of concentration, the highest ground-level O_3_ concentrations in Chonburi were around 70.5–74.6 ppb, which was lower than in China, where the levels were 81.5–91.7 ppb, observed as the highest in urban and suburban regions [21]. These disparities can be explained by regional variations in emissions, weather patterns, and urbanization that have a substantial impact on ozone dynamics. Furthermore, variations in ozone peaks, especially in metropolitan areas, are attributed to increased household VOC emissions and continuous NOx emissions during rush hours [21,27].

This aligns with the findings by Singleton et al. 2016, in which the higher humidity led to a steady increase in OH levels and a 67% reduction in O_3_ concentrations [28]. Moreover, high humidity often comes with rain; while this precipitation does not directly impact O_3_ concentrations, it often washes away soluble pollutants like nitric acid (HNO_3_) and hydrogen peroxide (H_2_O_2_) from the atmosphere. This removal, in turn, affects the availability of nitrogen oxides (NO_x_) and hydroxyl radicals (OH), which play a role in ozone formation and other atmospheric processes [14,29]. Consistently, a negative correlation was observed between the ozone and relative humidity during high particulate events in urban areas of Malaysia. This could be related to several atmospheric chemical reasons, such as ozone being more readily removed from the atmosphere in conditions with high humidity. However, a non-linear correlation was observed in a study from China, in which the maximum ozone concentration occurred in 55% RH. The relationship with global radiation showed similarities with the correlation value of 0.52 in a previous study from Lithuania [30]. This relationship is consistent with the understanding that sunlight triggers the photochemical reactions that produce ozone in the lower atmosphere, and O3 levels are significantly higher on clear days compared to cloudy days. This finding underscores the importance of global radiation in the formation of O3 [31]. Some studies have suggested non-linear interactions among photochemical reactions and significant seasonal variations influencing the overall correlation between ozone levels and temperature in the atmosphere [32]. The results aligned with previous research [33]; however, there was a very strong correlation between O_3_ and temperature in Malaysia [34]. The underlying reason would be that higher temperatures are capable of accelerating the chemical reactions that produce ozone. Some studies have indicated strong temperature-dependent reactions in surface O_3_ chemistry, evidenced by varying ozone generation rates with temperature changes [35]. According to Porter and Heald, the relationship of O_3_ and temperature contribute 3% to 9% in the U.S. and Europe. Temperature was affected by the emissions of volatile organic compounds (VOCs) and nitrogen oxides (NOx), which are critical for ozone formation. For instance, biogenic VOC emissions increase with the temperature. Moreover, the thermal decomposition of peroxyacetyl nitrate (PAN) accounts for approximately 20% [32]. Contradictorily, the O_3_ concentration was negatively correlated with wind speed in a study from Shanghai [36], and southwest and northeast monsoon winds play a major role in driving positive annual trends in O_3_-related chemical reactions, enhancing photochemical production [37].

However, particulate matter can provide surfaces for heterogeneous reactions, which may impact ozone levels. A study from mainland China indicated that the correlation between ozone and PM_2.5_ was positive in summer and negative in winter [38]. Nonetheless, the results contradict the evidence from a study in Northern Colombia in which ozone was negatively correlated with PM_10_ and PM_2.5_ at the values of −0.37 and −0.30, respectively [39]. On another hand, the correlations for other pollutants like CO and SO_2_ with O_3_ (−0.6 to −0.16 and 0.08 to 0.15) do not indicate strong relationships, indicating a weaker monotonic relationship at this location [40,41]. A negative correlation between the O_3_ and NO_2_ concentrations (0.26–0.36) was observed across all seasons [42,43], although this phenomenon is not widely understood, as several studies conducted in China and Bangladesh have indicated a positive correlation [40,44].

Nevertheless, our findings are similar, indicating O_3_ levels are known to rise with higher temperatures and GLRD, but interestingly, they also increase with higher GLRD, even at lower temperatures [1]. Global solar radiation exerts a significant influence on temperature and ozone levels in the tropical stratosphere. The solar impact on temperature exhibits positive correlations in the lower stratosphere and near the stratopause, while ozone responses vary significantly [45]. These observations align with the characteristics of O_3_, which also show that explanatory variables of GLRD and temperature are interrelated. However, O_3_ is produced through a photochemical reaction in the atmosphere at a location affected by the surrounding temperature, sunlight intensity, and the concentration of nitrogen oxides (NO_x_) [2]. Nevertheless, we found that the relationship between O_3_, humidity, and temperature is more erratic during this season. Lastly, the winter season figure shows a sharper gradient, akin to the dry season, but with a broader range of high ozone levels as the temperature and relative humidity increase. The O_3_ levels are at their highest during sunny days [46]. The relationship between ozone and the meteorological variables (temperature and relative humidity) was relatively low, and the explained variance was 34–48% for all seasons of the model. Moreover, it found a high contribution from the interaction of temperature with relative humidity (67–73%). The total ozone was analyzed from the TROPOspheric Monitoring Instrument (TROPOMI)/Sentinel-5 Precursor (S5P), combined with stratospheric ozone data [47]. The Getis–Ord Gi* spatial statistics method [48] was used to identify hotspots, highlighting areas where high ozone levels pose risks to public health and the environment. These hotspots, indicating high-risk areas, warrant urgent investigation and management to reduce ozone levels and mitigate health impacts. Conversely, cold spots, signifying low-risk areas, may reflect cleaner air or effective pollution control measures. A positive Moran’s I value with a 95% significance level [49] suggests that O_3_ concentrations are grouped together in Chonburi Province.

## 5. Conclusions

This study focused on the relationships between ground-level ozone concentrations and other climatic factors and pollutants, as well as the seasonal variations and temporal distributions of those concentrations in Chonburi, Thailand. According to the data, there was a distinct seasonal trend, with winter exhibiting the greatest O_3_ levels and rainy displaying the lowest, on average. The O_3_ concentrations in the dry seasons (winter and summer) were higher because of higher temperatures and penetrating sun radiation that encouraged photochemical O_3_ formation. However, while the O_3_ concentrations and solar radiation, temperature, and wind speed all showed high positive interactions, relative humidity displayed a substantial negative relation.

The highlight of this study was the significance of the interplay between temperature and other meteorological parameters, such as relative humidity, solar radiation, and wind speed, in influencing the ozone levels across different seasons. Additionally, the study applied generalized additive models (GAMs) to represent all coastal provinces. This approach provides important insights into ozone distribution, facilitating environmental management and planning to mitigate health and environmental impacts.

## Figures and Tables

**Table 1:** The estimate significance of smooth terms for different seasons in Chonburi Province.

	Dry Season				Rainy Season				Winter Season			
	EDF	Ref.df	F	*p*-Value	EDF	Ref.df	F	*p*-Value	EDF	Ref.df	F	*p*-Value
s(NO_2_)	6.261	7.311	70.175	<0.001	8.051	8.673	67.611	<0.001	5.276	6.373	145.092	<0.001
s(Temp)	8.206	8.803	12.668	<0.001	8.075	8.739	25.651	<0.001	8.301	8.838	16.666	<0.001
s(RH)	7.224	8.122	38.097	<0.001	8.229	8.749	50.089	<0.001	8.247	8.721	111.841	<0.001
s(Glrd)	8.850	8.991	45.190	<0.001	6.178	7.271	53.426	<0.001	8.805	8.987	47.643	<0.001
s(Ws)	6.363	7.526	45.428	<0.001	8.893	8.995	45.214	<0.001	8.013	8.625	16.766	<0.001
s(Temp, RH)	8.529	8.882	20.788	<0.001	8.087	8.735	89.947	<0.001	8.151	8.722	47.142	<0.001
s(Temp, Glrd)	8.910	8.994	49.786	<0.001	8.934	8.997	49.986	<0.001	8.916	8.995	54.034	<0.001
s(Temp, Ws)	7.757	8.397	52.662	<0.001	7.084	7.923	66.693	<0.001	7.704	8.391	29.705	<0.001
Deviance explained		50.9%					47.9%				53.7%	

EDF, equivalent degrees of freedom; F, test statistics; T, temperature; GLRD, global solar radiation; RH, relative humidity; WS, wind speed.

## Data Availability

Data sharing was not relevant to this study, since no new data were generated or examined in this study. The primary sources of all the data were presented in the ‘Data Source’ section.

## Supplementary Materials

The following supporting information can be downloaded at https://www.mdpi.com/article/10.3390/toxics13030226/s1: Figure S1: Time variation plot of the air quality (NO_2_, SO_2_, PM_2.5_, and PM_10_); Figure S2: Time variation plot of the air quality (GLRD, rain, Rh, WS, and WD.); Figure S3: Variation in the annual surface wind direction on the coast of Chonburi Province (a–d) by season; Table S1: Descriptive statistics across different seasons or times of the day.

## Abbreviations

O_3_	Ozone
SO_2_	Sulfur dioxide
NO_x_	Nitrogen oxides
HNO_3_	Nitric acid
VOCs	Volatile organic compounds
T	Temperature
GLRD	Global solar radiation
RH	Relative humidity
WS	Wind speed
WD	Wind direction
GAM	Generalized additive models
