# Supplementary material for: The Influence of Meteorological Conditions and Seasons on Surface Ozone in Chonburi, Thailand
Source: Toxics. 2025 Mar 19;13(3):226. doi: 10.3390/toxics13030226 (PMC11946029; doi:10.3390/toxics13030226)
Supplement: Supplementary file 1 [file toxics-13-00226-s001.zip › toxics-3468423-supplementary.pdf]

Supporting Information

Table S1. Descriptive statistics across different seasons or times of the day.

Parameter	Season	count	mean	std	min	25%	75%	max
O_3	Dry	23476.00	24.89	17.04	0.30	12.23	33.33	138.50
(ppb)	Rainy	40329.00	18.74	13.19	0.20	9.67	23.90	145.00
	Winter	32369.00	26.90	18.10	0.20	12.67	37.53	146.50
NO_2	Dry	23385.00	12.48	7.06	0.33	7.67	15.50	76.33
(ppb)	Rainy	40261.00	11.26	6.52	0.50	7.00	13.77	85.05
	Winter	32380.00	14.60	9.03	0.33	8.00	19.00	101.00
SO_2	Dry	18501.00	2.69	2.81	0.10	1.00	3.33	78.20
(ppb)	Rainy	34510.00	2.82	2.92	0.05	1.00	3.50	81.70
	Winter	27007.00	2.48	2.37	0.05	1.00	3.00	87.70
CO	Dry	21486.00	0.60	0.30	0.05	0.40	0.70	2.60
(ppm)	Rainy	35404.00	0.54	0.28	0.05	0.40	0.64	3.20
	Winter	28534.00	0.66	0.32	0.05	0.43	0.80	2.80
PM_{10}	Dry	23560.00	36.06	20.51	1.00	21.67	45.67	205.50
(mg/m3)	Rainy	40384.00	28.61	17.59	1.00	17.00	35.33	218.40
	Winter	32403.00	40.65	23.45	1.00	24.00	51.67	248.33
$PM_{2.5}$	Dry	11347.00	22.17	13.28	0.80	13.00	28.00	102.10
(µg/m^3)	Rainy	20613.00	15.70	11.31	0.70	9.00	19.00	170.60
	Winter	16357.00	24.89	15.98	0.70	13.33	33.00	161.00
	Dry	23568.00	28.78	3.15	17.60	26.50	31.03	39.77
Temperature	Rainy	40392.00	29.01	2.84	16.57	27.03	30.90	40.13
(°C)	Winter	32472.00	28.15	3.43	15.03	25.70	30.53	48.70
	Dry	21352.00	72.23	13.94	17.00	63.00	83.00	99.00
Relative	Rainy	34318.00	73.75	13.18	1.00	65.00	83.33	99.33
Humidity								
(%)	Winter	28408.00	69.03	15.93	1.00	58.00	81.00	99.50
	Dry	11261.00	758.48	3.16	744.50	756.67	760.67	767.00
Pressure	Rainy	18710.00	757.57	16.83	746.33	755.67	759.00	767.00
(mmHg)	Winter	15524.00	759.18	2.87	749.67	757.33	761.00	768.00

	Dry	22641.00	194.03	292.36	0.00	0.00	320.00	1449.00
Glrd	Rainy	39033.00	194.74	292.52	0.00	0.00	312.00	1373.00
(w/m2)	Winter	31209.00	185.44	286.09	0.00	0.00	292.00	1446.00
	Dry	18578.00	0.15	1.47	0.00	0.00	0.00	74.00
Rain	Rainy	29630.00	0.20	1.77	0.00	0.00	0.00	93.33
(mm)	Winter	23830.00	0.11	1.07	0.00	0.00	0.00	45.00
	Dry	23568.00	1.59	0.88	0.00	0.93	2.10	6.85
Ws	Rainy	40392.00	1.60	0.87	0.04	0.93	2.10	6.60
(m/s)	Winter	32472.00	1.36	0.78	0.00	0.77	1.80	6.40
	Dry	23568.00	163.52	60.09	0.00	126.00	204.00	357.00
Wd	Rainy	40392.00	181.59	61.13	6.00	143.00	220.00	358.00
	Winter	32472.00	144.22	75.34	3.00	76.50	199.67	357.33

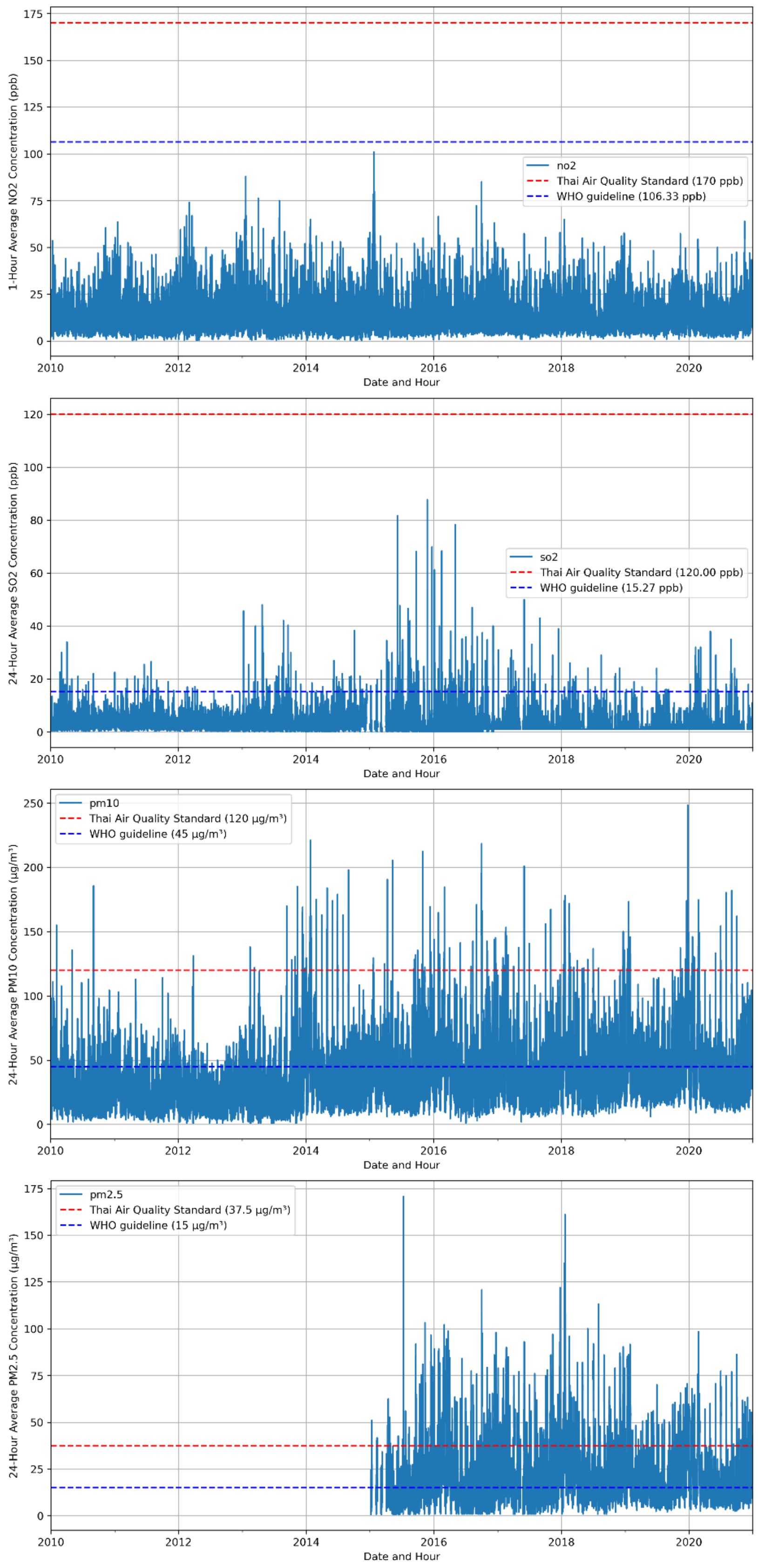

Figure S1. Time variation plot of air quality (NO_2, SO_2, $PM_{2.5}$, PM_{10})

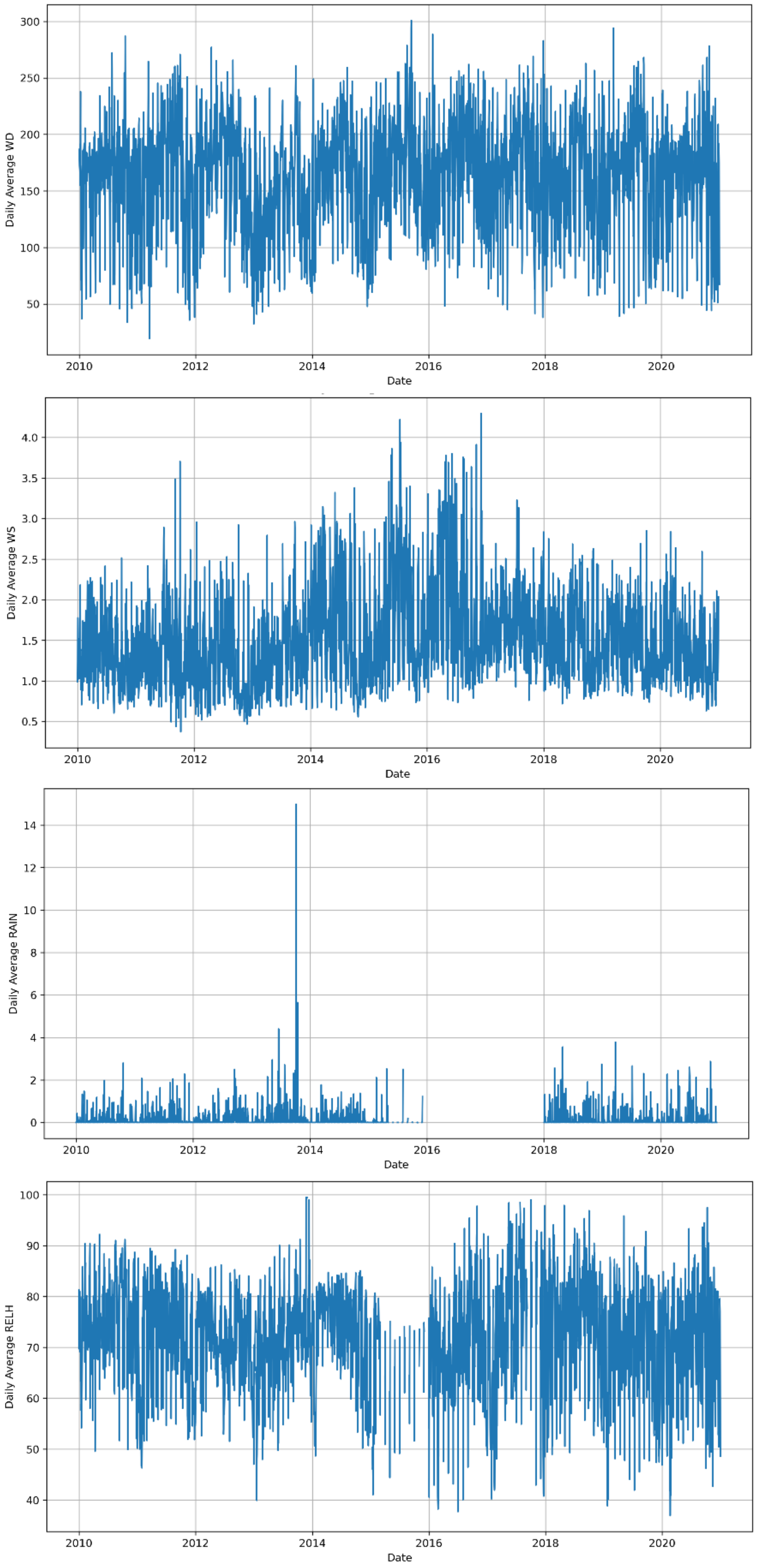

Figure S2. Time variation plot of air quality (Glrd, rain, Rh, WS and WD.)

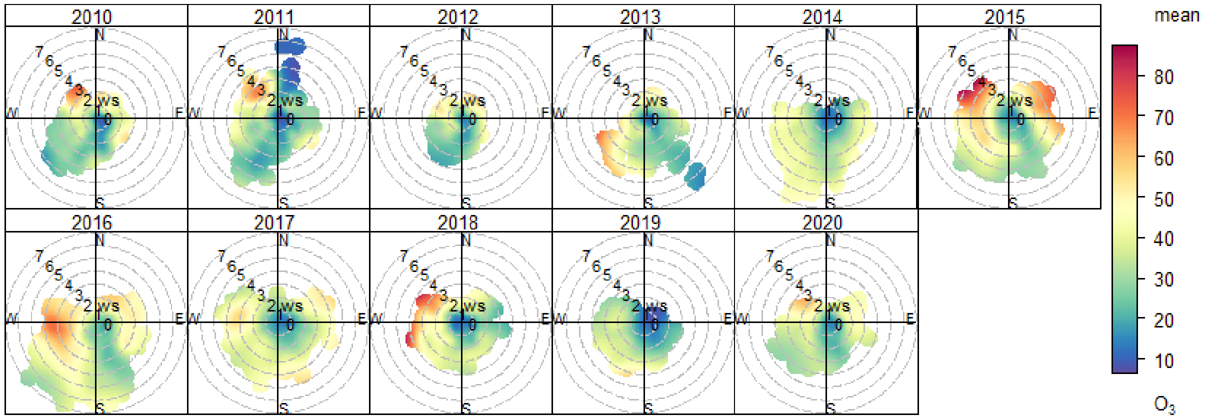

(a) Dry season

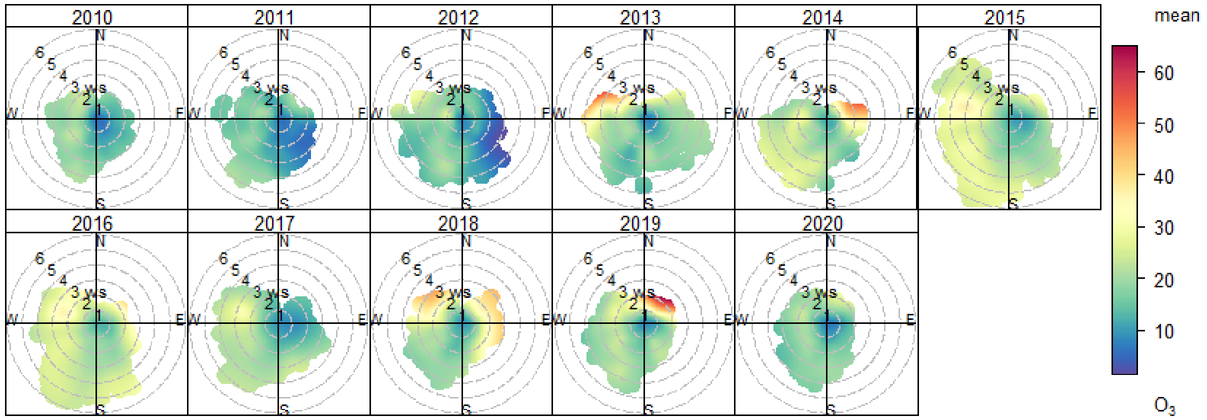

(b) Rainy season

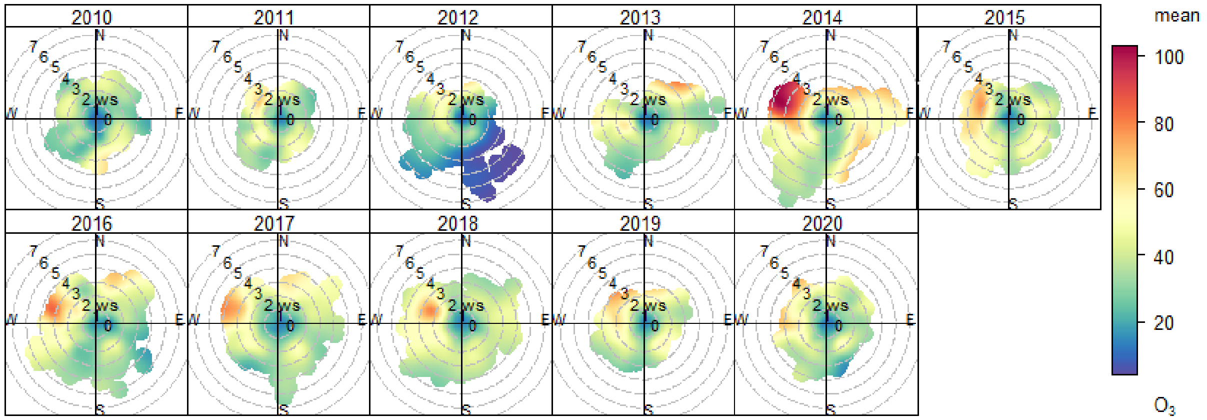

(c) Winter Season

Figure S3. Variation in annual surface wind direction in costal of Chonburi province (a-d) by season
